# Anti-Androgen Receptor Therapies in Prostate Cancer: A Brief Update and Perspective

**DOI:** 10.3389/fonc.2022.865350

**Published:** 2022-03-10

**Authors:** Jian Huang, Biyun Lin, Benyi Li

**Affiliations:** ^1^ Pathological Diagnosis and Research Center, The Affiliated Hospital of Guangdong Medical University, Zhanjiang, China; ^2^ Department of Urology, The University of Kansas Medical Center, Kansas City, KS, United States

**Keywords:** androgen receptor, prostate cancer, small interfering RNA, protein degradation, PROTAC

## Abstract

Prostate cancer is a major health issue in western countries and is the second leading cause of cancer death in American men. Prostate cancer depends on the androgen receptor (AR), a transcriptional factor critical for prostate cancer growth and progression. Castration by surgery or medical treatment reduces androgen levels, resulting in prostatic atrophy and prostate cancer regression. Thus, metastatic prostate cancers are initially managed with androgen deprivation therapy. Unfortunately, prostate cancers rapidly relapse after castration therapy and progress to a disease stage called castration-resistant prostate cancer (CRPC). Currently, clinical treatment for CRPCs is focused on suppressing AR activity with antagonists like Enzalutamide or by reducing androgen production with Abiraterone. In clinical practice, these treatments fail to yield a curative benefit in CRPC patients in part due to AR gene mutations or splicing variations, resulting in AR reactivation. It is conceivable that eliminating the AR protein in prostate cancer cells is a promising solution to provide a potential curative outcome. Multiple strategies have emerged, and several potent agents that reduce AR protein levels were reported to eliminate xenograft tumor growth in preclinical models *via* distinct mechanisms, including proteasome-mediated degradation, heat-shock protein inhibition, AR splicing suppression, blockage of AR nuclear localization, AR N-terminal suppression. A few small chemical compounds are undergoing clinical trials combined with existing AR antagonists. AR protein elimination by enhanced protein or mRNA degradation is a realistic solution for avoiding AR reactivation during androgen deprivation therapy in prostate cancers.

## Introduction

Prostate cancer is the second most common type of cancer diagnosed in men worldwide and the second leading cause of male cancer-related deaths in the U.S. (1). The American Cancer Society estimates about 268,490 new cases of prostate cancer and about 34,500 deaths from prostate cancer in the U.S. this year (1). According to the American Cancer Society data (cancer.org), patients with local or regional stage prostate cancer have nearly a 100% 5-year survival rate; however, the survival rate is only 30% for men diagnosed with distal metastasis.

**Figure 1:**
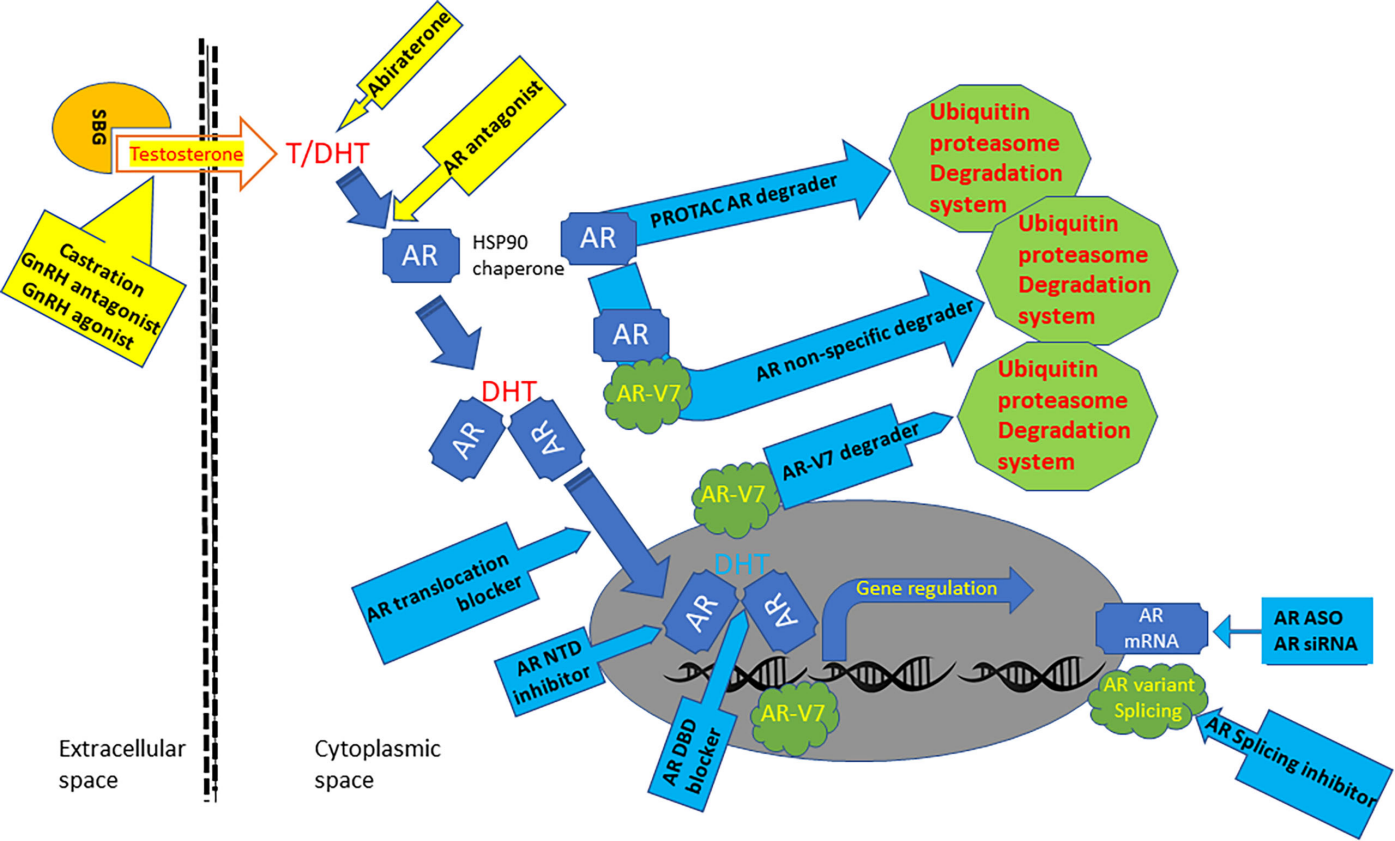
Graphic scheme of AR-targeted agents. Androgens are bonded with steroid-binding globulins (SBG) in the bloodstream for systemic circulation. Androgen testosterone (T) is converted to potent form dihydrotestosterone (DHT) in the cytoplasm by 5a-reductase. The AR protein bonds with HSP90 chaperones and resides in the cytoplasmic compartment before androgen binding. Androgen binding alters AR conformation and promotes its translocation into the nuclear compartment, where it interacts with chromatin DNA to regulate gene expression. AR gene mRNA is aberrantly spliced in advanced prostate cancers to generate variant proteins like AR-V7, which is constantly active without androgen binding. Current clinical therapies for metastatic prostate cancers (yellow background box) include castration, GnRH agonist and antagonist, Abiraterone, and AR antagonists. Several AR-targeted treatments under development (blue background box) include AR PROTAC and non-specific degraders, AR-V7 degraders, AR-NTD inhibitor, AR-DBD blocker, AR nuclear translocation blockers, AR splicing inhibitors.

Currently, localized prostate cancer is primarily treated with surgical removal of the gland or radiation therapy if a patient’s condition is not permissive for surgery. Distal metastasis occurs in high-risk patients, including locally advanced (positive surgical margin) or high-grade (Gleason sum score ≥ 8) tumors, which is the sole cause of death from prostate cancer (2). This short review work will discuss the current treatment options and recent development of anti-androgen receptor (AR) therapeutic approaches for metastatic prostate cancer (**Table 1** and **Figure 1**).

**Table 1:** Summary of AR-targeted therapeutic agents for prostate cancers.

Therapeutic Target	Agent Or Approach	Mechasnism Of Action	Current Stage	Reference
**Testicular androgens**	surgical castration	testis removal	in clinic use	(3)
	GnRH antagonist	reducing testersterone production	in clinic use	(4)
	GnRH agonist	reducing testersterone production	in clinic use	(4)
**Adrenal or cancer androgens**	Abiraterone	CYP17A1 inhibition	in clinic use	(5)
**all androgens**	Flutamide	blocking androgen-AR binding	in clinic use	(5)
	Bicalutamide	blocking androgen-AR binding	in clinic use	(5)
	Enzalutamide	blocking androgen-AR binding	in clinic use	(5)
	Apalutamide	blocking androgen-AR binding	in clinic use	(5)
	Darolutamide	blocking androgen-AR binding	in clinic use	(5)
**AR mRNA**	antisense oligonucleotides	mRNA-based protein translation and mRNA stability	pre-clinical	(6–14)
	small interfering RNA	mRNA silencing	pre-clinical	(15–23)
**Full length AR protein**	ARCC-4/ARV-110	PROTAC-mediated AR degradation	phase-1 clinical trial	NCT03888612
	ARD series	PROTAC-mediated AR degradation	pre-clinical	(24–31)
	TD-802	PROTAC-mediated AR degradation	pre-clinical	(32)
	A031	PROTAC-mediated AR degradation	pre-clinical	(33)
	MTX-23	PROTAC-mediated AR degradation	pre-clinical	(34)
	A9/A16	PROTAC-mediated AR degradation	cell culture model	(35, 36)
	SNIPER-51	PROTAC-mediated AR degradation	cell culture model	(37)
**Full-length/variant AR protein**	UT-34	AR NTD binding and degradation	pre-clinical	(38)
	Ailanthone	co-chaperone p23 binding and AR degradation	pre-clinical	(39)
	HG122	proteasome-based AR degradation	pre-clinical	(40)
	CUDC-101	AR degradation due to unknown mechanism	pre-clinical	(41)
	ASC-J9	AR degradation due to unknown mechanism	pre-clinical	(42–47)
**AR splicing variants**	Niclosamide	AR-V7 degradation	phase-1 clinical trial	NCT03123978
	Niclosamide	AR-V7 degradation	phase-1 clinical trial	NCT02807805
	Thailanstatins	suppressing splicing event for AR-V7	pre-clinical	(48–50)
	Rutaecarpine	AR-v7 degradation via GPR78/SIAH2 pathway	pre-clinical	(51)
	Indisulam	Suppressing AR-V7 splicing factor RBM39	pre-clinical	(52)
	Nobiletin	AR-V7 degradation via blocking USP14/USP22	pre-clinical	(53)
**AR NTD inhibitor**	EPI series/EPI-7386	suppressing AR NTD TAU-5 activity	phase-1/2 clinical trial	NCT05075577
	EPI series/EPI-7387	suppressing AR NTD TAU-5 activity	phase-1 clinical trial	NCT04421222
	QW07	suppressing AR NTD activity	pre-clinical	(54)
**AR nuclear translocation**	EPPI/CPPI	blocking AR nuclear translocation	pre-clinical	(55–57)
	IMPPE	blocking AR translocation and inducing AR degradation	pre-clinical	(58)
	JJ-450	blocking AR translocation and transactivation	pre-clinical	(59–62)
**AR DND-hinge antagonist**	VPC-14228/14449	blocking AR dimerization and DNA binding	pre-clinical	(63–66)

## Androgen Deprivation and Anti-Androgen Therapies in the Clinic

Metastatic prostate cancers are initially treated with androgen deprivation therapy (ADT) because prostate tissue (benign or malignant) expresses androgen receptor (AR) protein that is critical for prostate cancer development and progression (3, 4). Castration by surgery or medical treatment reduces androgen hormones, resulting in prostatic atrophy and cancer regression (5). This approach was developed eighty years ago in 1941 (3, 4). Since then, prostate cancer treatment has been mainly focused on reducing androgen levels and blocking androgen-induced AR activation (5). However, prostate cancers often relapse and progress to a stage termed as castration-resistant prostate cancers (CRPC) (67, 68), and the majority of these CRPCs still depend on the AR signaling for growth and progression (the AR addictiveness) (69, 70).

The mechanisms for CRPC progression include AR gene mutation, amplification, transcriptional splicing, and crosstalks with cellular signal pathways, plus *de novo* androgen synthesis by the malignant prostate cells (5). Therefore, clinical therapies use anti-androgens (Flutamide, Bicalutamide, Enzalutamide, Apalutamide, and Darolutamide) to competitively suppress androgen-induced AR activation or CYP17A1 inhibitor (Abiraterone) to reduce androgen production in prostate cancer tissues (5). So far in the clinic, these therapies provided certain clinical benefits of survival extension in CRPC patients (71). However, with the widespread use of Enzalutamide and Abiraterone, a subset of CRPC patients developed neuroendocrine progression, termed as anti-AR treatment-induced NEPC (t-NEPC) (72, 73), accounting for more than 25-30% mortality of CRPC fatality (74). There were multiple mechanisms involved in NEPC progression, including attenuated control of transcriptional factors, metabolic alterations, aberrant activation of cellular kinases, long noncoding RNAs, transcriptional splicing, and epigenetic modifications (75–87). It is postulated that extensive stress of AR inhibition under the long-term ADT condition forced an epigenetic reprogramming of CRPC cells into neuroendocrinal trans-differentiation (88–93). Treatment option for NEPC patients is limited in the clinic and the salvage platinum-based chemotherapy only provided very little survival benefit (75).

## AR Protein Elimination Approaches in Preclinical Development Phase

The AR protein is a nuclear receptor expressed in benign and malignant prostate tissues, critical for prostate physiological functionality and prostate cancer progression (94, 95). As a transcriptional factor, the AR protein modulates gene expression after being activated by androgens *via* binding on its C-terminal ligand-binding domain (95). Given that hormone therapy, including ADT and anti-androgens for the last eighty years, has been failed to be a curable approach for metastatic prostate cancers, eliminating the AR protein in prostate cancer cells recently emerged as a realistic solution for a potentially curable result.

### Antisense Oligonucleotide Technology

Antisense oligonucleotides (ASOs) are synthetic complementary single-stranded deoxyribonucleotides used to target messenger RNA (mRNA) of targeted genes, resulting in RNase H endonuclease-dependent mRNA cleavage or blockage of protein translation (6). Dr. Klocker’s group reported the first study using the ASO technology against the AR gene in 2000, which showed a suppressive effect on prostate cancer LNCaP cell growth (7). A follow-up study by the same group showed the *in vivo* effectiveness of suppressing LNCaP-derived xenograft tumors in nude mice (8). These initial results were supported by the studies from other groups (9, 10). Possibly due to the suppressive nature of ASOs on target gene expression, the AR protein was not eliminated from cancer cells. Also, the results only showed a moderate suppressive effect on tumor growth because of the difficulty in tissue delivery of the ASO molecules. However, these AR-targeted ASOs showed an enhanced effect when combined with other gene targets (EZH2 or Clusterin) for Enzalutamide-resistant CRPC models (11–14). A recent report achieved a successful *in vivo* delivery of AR-specific ASO using lipid-based nanotechnology. A profound suppressive effect was achieved in the prostate cancer xenograft model, together with a significant reduction of the AR protein levels in xenograft tumor tissues (96).

### Small Interfering RNA Technology

Since the introduction of small interfering RNA (siRNA) technology in 2001 (97, 98), knocking down gene expression in living organisms became possible. To overcome the clinical obstacle of anti-AR treatment resistance, we hypothesized that eliminating AR protein from prostate cancer cells might completely shut down AR signaling, leading to cell death or growth arrest. Knocking down AR gene expression in prostate cancer cells resulted in profound apoptotic cell death in multiple prostate cancer cell lines, androgen-responsive or castration-resistant (15). Nanoparticle-based prostate cancer-specific delivery approach and adenoviral approach to systemically deliver the AR siRNA expression particles documented a rapid xenograft tumor regression and eradication owing to robust cell death *in vivo* (16, 17). These findings were overwhelmingly supported by reports from other groups using divergent approaches to knock down AR gene expression (18–23). These results confirmed that eliminating AR protein (full length or truncated) will overcome treatment resistance in advanced prostate cancers.

### PROTAC Technology

PROTAC stands for proteolysis targeting chimera. It uses a small bifunctional molecule with two binding moieties connected by a linker to bring together a targeted protein and cellular proteolytic machinery, ubiquitin E3 ligase-mediated proteasome degradation system (99, 100). This technology selectively removes specific proteins like the AR protein for a therapeutic purpose (101, 102). Several descent review articles summarized the technique description and the usage of various E3 ligases (103–106). We will only discuss the PROTAC molecules designed for the AR protein.

The first AR-targeted PROTAC approach was reported in 2004, which used a synthetic peptide targeting the E3 ligase fused to either an artificial FKBP12 ligand or dihydrotestosterone (DHT) (24). After several optimizations, a potent AR-specific PROTAC molecule ARCC-4 was developed with a nanomole concentration efficiency (25). Its further modified version, ARV-110, is being tested in clinical trials in metastatic prostate cancer patients (26). The first trial is a phase-1b open-label clinical trial (NCT05177042) to assess the combination of ARV-110 and Abiraterone in patients with metastatic prostate cancer with PSA progression after Abiraterone treatment. It is estimated to finish at the end of April of 2023. The second one is a phase-1/2 open-label single-agent dose escalation and cohort expansion trial to assess the safety and tolerability of ARV-110 (NCT03888612). It will be finished at the end of February 2023.

The AR degrader (ARD) series of PROTAC molecules (ARD-61, -69, -266, -2128, -2585) were reported from Dr. Wang’s group at the University of Michigan (27–31). Their latest molecule, ARD-2585, is a potent (DC_50_ < 0.1 nM) oral agent and has at least 10-fold more potent than ARV-110 (27). These molecules differ in distinct E3 ligase binding domains, AR antagonists, and variable lengths of the linkers. Unfortunately, both ARV-110 and ARD-2585 molecules depend on binding with the AR LBD. Therefore, it is not effective on the AR splicing variants like AR-V7.

Other AR-targeted PROTAC molecules with animal testing data include TD-802 (DC_50_ = 12.5 nM) (32) and A031 (IC_50_ < 0.25 μM) (33) that promote degradation of the full-length AR protein. MTX-23 was shown to promote protein degradation of both the full-length and AR-V7 variant AR protein (DC_50_ = 0.37-2 μM) (34). In addition, three PROTAC molecules, A9/A16 (35, 36) and AR SNIPER-51 compounds (37), were only tested in cell culture models.

### Other Unique Molecules for AR Degradation

UT-34 is a small molecule that exerts potent AR degradation activity *in vitro* (1-10 μM) and *in vivo via* ubiquitin-proteaseom pathway (38). It was optimized from its two previous versions, UT-69 and UT-155 (107). UT-34 binds with the AR N-terminal AF-1 domain and thus targets both the full-length and splicing variant proteins. UT-34 has a good pharmacological profile of oral bioavailability and suppressed xenograft tumor growth derived from Enzalutamide-resistant prostate cancer cells at a dose of 60 mg/kg/day (38).

Ailanthone was initially identified as an inhibitor of AR transactivation *via* a high throughput screening assay and was later found to induce protein degradation of both full-length and splicing variant AR proteins *via* targeting an HSP90 co-chaperon protein p23 (39). Ailanthone exhibited a strong anti-cancer effect in both *in vitro* cell culture models (0.2-0.4 μM) and *in vivo* xenograft models (2 mg/kg/day) of prostate cancer (39). It also showed excellent drug-like properties as tested in preclinical models (108, 109).

HG122 was identified as an inhibitor of AR activity *via* an MMTV-luciferase assay-based high throughput screening (40). HG122 suppressed AR-positive prostate cancer cell growth with an IC_50_ of 7-9 μM, compared to AR-negative cells at 20 μM. HG122 suppressed AR transcriptional activity and promoted AR degradation *via* the proteasome pathway. In animal experiments, HG122 suppressed 22RV1 cell-derived xenograft tumor growth by 82% at a dose of 10 mg/kg/day, compared to a 60% reduction by Enzalutamide at the exact dosing (40). However, it is unclear how HG122 promoted AR degradation by the proteasome machinery.

### AR Splicing Variant V7-Specific Degraders and Inhibitors

The full-length AR protein has four distinct domains, N-terminal (NTD), DNA-binding (DBD), hinge region, and C-terminal ligand-binding (LBD). In prostate cancers, the transcriptional splicing variants of the AR gene have been linked to castration-resistance of prostate cancer after ADT and anti-AR therapy with Enzalutamide and Abiraterone (110–112). Because these AR variant proteins lack the AR C-terminal LBD region due to gene splicing truncated or deleted, they are not responding to current anti-AR drugs that target the LBD. Therefore, those PROTAC molecules using the LBD ligands are not working on these splicing variant AR proteins (113–115). These variant proteins represent a massive obstacle to clinical management in advanced prostate cancers.

Niclosamide is an FDA-approved oral anti-helminthic drug used to treat parasitic infections. In an AR-V7-driven luciferase-based high-throughput screening assay, Niclosamide was identified as an effective inhibitor of AR-V7 activity. A mechanistic study showed that it enhanced the AR-V7 protein degradation *via* the ubiquitin-proteasome pathway in prostate cancer cells at 0.5-1.0 μM without affecting the full-length AR protein (116). Combinational treatment with Enzalutamide and Niclosamide suppressed CRPC xenograft tumor growth in mice at a dose of 25 mg/kg/day (117). Although the first clinical trial (NCT02532114) with a single dose of Niclosamide was failed in reaching the effective serum concentration (118), a recent phase-Ib trial with reformulated Niclosamide plus Abiraterone achieved the proposed clinical benefit (119), representing a new hope for AR-V7 positive CRPC patients (NCT03123978/NCT02807805).

CUDC-101 is a small molecule of inhibitor for multiple targets, including histone deacetylase (HDAC), epidermal growth factor receptor (EGFR) and HER2/Neu. It was recently found to inhibit the transcriptional activities of the full-length AR and AR-v7 protein (0.3 μM for 24 h) *via* a HDAC-related mechanism in prostate cancer 22RV1 cells (41). It also suppressed 22RV1 cell-derived xenograft tumor growth in nude mice at a dose of 50 mg/kg/day for 14 days (41). However, severe side effects will be expected in a clinical test due to its action on multiple targets.

ASC-J9 is a curcumin analog (dimethyl-curcumin) with multiple protein targets (120–125), including the AR proteins (42–44). ASC-J9 induced protein degradation of the full-length AR and AR-V7 proteins *via* the ubiquitin-proteasome pathway in prostate cancer cells (44) and suppressed xenograft tumor growth derived from CRPC cells (42, 45). It overcame Enzalutamide resistance in preclinical CRPC xenograft models (46) and sensitized prostate cancers to radiation therapy in animal models (47). However, ASC-J9 was only tested in clinical trials for skin acne care (NCT01289574 and NCT00525499).

Thailanstatins are bacteria-derived natural products with potent inhibitory activity toward pre-mRNA splicing events (48). Since AR-V7 is mainly generated by pre-mRNA splicing (49), Thailanstatin D (TST-D) was tested in AR-V7 positive prostate cancer cells for cytotoxicity. TST-D was shown to reduce AR-V7 mRNA and protein levels (at 5 nM concentration) by disrupting the U2AF65/SAP155 splicing complex that is critical for the AR-V7 pre-mRNA expression and suppressed CRPC cell-derived xenograft tumor growth (50% inhibition at 0.3 mg/kg/day after four days) (50). It is postulated that combinational treatment of TST-D with Enzalutamide or Abiraterone might achieve a more profound anti-tumor effect in CRPC models.

Rutaecarpine is a cardiovascular protective alkaloid extracted from the Chinese medicine *Evodia rutaecarpa* (126). It was identified as a potent AR-V7 inhibitor in an AR-V7-driven luciferase screening assay (51). A mechanistic study revealed that Rutaecarpine promoted AR-V7 degradation by enhancing AR-V7 interaction with GPR78 and ubiquitin E3 ligase SIAH2. Its DC_50_ for AR-V7 degradation was about 20 μM and completely blocked 22RV1 cell-derived xenograft tumor growth in nude mice at 40 mg/kg/2day (51). Since it also did not affect the full-length AR protein, it is needed to test its synergistic effect with AR antagonists like Enzalutamide and Abiraterone *in vivo*.

Indisulam belongs to a new class of compound sulfonamide with potential antineoplastic activity (127) *via* selectively degrading oncogenic proteins like pre-mRNA splicing factor RBM39 (52). Because pre-mRNA splicing is critical for AR-V7 expression, Indisulam was shown to suppress AR-V7 expression *via* RBM39-dependent mechanism. Indisulam treatment blocked Enzalutamide-induced AR-V7 expression in VCaP cells (10 μM concentration) and suppressed VCaP cell-derived xenograft tumor growth in nude mice at a dose of 25 mk/kg/day (52).

Nobiletin is a plant flavonoid extracted from *citrus peels* and possesses broad anti-cancer activity (128, 129). A recent study showed that Nobiletin moderately reduced AR-V7 protein level in 22RV-1 cells at 20 μM concentration and synergistically suppressed (at 40 mg/kg/2day) 22RV1 cell-derived xenograft tumor growth with Enzalutamide (20 mg/kg/2day) (53). The mechanistic study revealed that Nobiletin disrupted AR-V7 interaction with two deubiquitinases, USP14 and USP22, leading to proteasome-based AR-V7 degradation (53).

## AR N-Terminal Specific Inhibitors

In contrast to the CTD, the AR NTD has very few mutations without truncation (130). For example, the cBioportal database showed only 9 (0.145%) point-mutations identified from the NTD regions in 6334 prostate cancer specimens. There are two transactivation unit (TAU-1, aa100-370) and TAU-5 (aa360-485) motifs within the AR NTD (131). The TAU-1 motif is critical for the full-length AR activation after ligand binding, while the TAU-5 motif functions as a constitutive active motif for truncated AR protein (e.g., AR-V7) (132, 133). Especially, the TAU-1/TAU-5 motifs are rarely mutated or deleted in prostate cancer patients, making them a feasible target for prostate cancer therapy (130).

EPI series compounds are the first class of AR NTD inhibitors. The first compound EPI-001 was identified by screening a library of marine sponge extracts to inhibit AR NTD transactivation activity (134). EPI-001 binds to the TAU-5 motif and inhibits AR NTD activity at a relatively high dose (>25 μM in cell culture models) (135, 136). EPI compounds also suppressed tumor growth in VCaP and LNCaP95 cell-derived xenograft models at 100-200 mg/kg/day doses (135, 137). Although the older EPI compounds did not affect AR protein levels (the full length and AV variants), the new analog EPI-7170 suppressed AR-V7 expression in CRPC cells (138). EPI-002 (commercial name Ralaniten) is one of the four EPI-001 stereoisomers, and its pro-drug EPI-506 (Ralaniten acetate) was failed in a phase-I clinical trial due to excessive pill burden and poor oral bioavailability (139, 140). The newest analog, EPI-7386, showed 20-fold higher anti-androgenic potency than Ralaniten (141), and it is being tested in clinical trials in combination with Enzalutamide (NCT05075577/NCT04421222).

QW07 is a small synthetic molecule identified as an AR NTD-specific inhibitor *via* an AR-NTD-driven luciferase high-throughput screening (54). QW07 suppressed the activity of AR full-length and splicing variants at 5-8 μM in prostate cancer cells, which is more potent than EPI-001 (54). QW07 binds with the AR NTD directly and suppresses AR recruitment onto the target gene promoter. In animal xenograft experiments, QW07 inhibited tumor growth derived from prostate cancer 22RV1 and VCaP cells at a dose of 40 mg/kg/day, similar to EPI-001. However, QW07 did not affect AR protein expression (the full length or splicing variants).

## AR Nuclear Translocation Blockers

As a transcription factor, the AR proteins translocate into the nuclear compartment after being activated by the androgens (5). In the nuclear, AR protein interacts with the androgen response elements in the gene promoter region to modulate gene expression. The AR protein has one nuclear localization sequence or signal (NLS) in each domain, the NTD region (aa294-556), the DBD-hinge region (aa617-633), and the LBD region (aa666-919) (142–144). In the absence of androgens, the AR protein is exported from the nuclear compartment *via* its nuclear export signal (NES, aa743-817) within the LBD region (145). In CRPC tissue or cells that androgen levels are deficient due to androgen deprivation therapy, the NLS in the NTD region is responsible for AR nuclear localization (143). Blocking AR nuclear translocation with a potent NLS inhibitor is feasible to suppress prostate cancer development and progression by shutting down AR-modulated gene expression.

EPPI and CPPI are small molecules identified as inhibitors of AR nuclear translocation in Dr. Z Wang’s lab using a 2GFP-AR fusing protein-based high-throughput screening approach (55). Both EPPI and CPPI at 25 μM inhibited AR nuclear localization in prostate cancer cells, which was reversed when the androgen level (R1881) was over 1.0 nM level, a physiological androgen concentration (56). Also, CPPI at a 50 mg/kg/day dose suppressed tumor growth in LNCaP but not PC-3 cell-derived xenograft models with or without castration, indicating an AR-specific effect (56). Further analysis revealed that CPPI blocked AR nuclear import and promoted AR degradation in the nuclear compartment through MDM2-dependent proteasome mechanism in CRPC cells (C4-2 and LNCaP95) and xenograft tumor models, leading to sharp retardation of tumor growth (57). No effect was observed for CPPI or EPPI on the AR variant proteins (57).

IMPPE (SID3712502) was another small molecule identified from the 2GFP-AR fusing protein screening assay with a robust inhibitory effect at 2.0 μM concentration on AR nuclear translocation and its downstream target PSA gene expression, plus downregulation of AR gene expression at a higher concentration of 10 μM (55). Further study found that IMPPE inhibited both full-length and LBD-lacking AR activity at a relatively high dose (>10 μM) and suppressed 22RV1 but not PC-3 cell-derived xenograft tumor growth at a dose of 25 mg/kg/day in castrated nude mice (58).

JJ-450 is an IMPPE scaffold analog with higher potency and better physicochemical properties (59). JJ-450 at 10 μM concentration inhibited both the transcriptional activities of the full-length and splicing variant AR proteins in CRPC cells by blocking AR binding to its target gene promoter without affecting AR protein levels (59). In CRPC xenograft models derived from 22RV1 and VCaP cells, JJ-450 at 10 mg/kg/day dose suppressed xenograft tumor growth by 60%, slightly better than Enzalutamide (59). Especially, JJ-450 was found to block the nuclear translocation and activity of the AR F876L mutant protein identified from Enzalutamide-resistant CRPC patients and LNCaP cells after long-term exposure to Enzalutamide (60–62).

## AR DBDH Antagonists

The AR DBD-Hinge region has P-box and D-box motifs responsible for dimerization and DNA binding after androgen stimulation (146). Using a virtual *in-silico* drug design approach (63–65), a surface-exposed region (aa579-610) on the AR DBDH domain was discovered as a potential target site by small-molecule compounds, including VPC-14228 and VPC-14449 (66). These two compounds at 10 μM concentration selectively suppressed AR (full-length and splicing variant proteins) but not ER or GR activity by blocking AR interaction with the target gene promoters without affecting AR nuclear translocation and protein stability (66). In LNCaP cell-derived xenograft experiments, VPC-1449 at 100 mg/kg/day dose suppressed tumor growth at a similar extent as Enzalutamide (10 mg/kg/day) (66).

## Conclusion and Perspectives

The AR protein is critical for prostate cancer progression by transcriptionally modulating gene expression after activation by androgens *via* binding on its LBD. Metastatic prostate cancers are initially treated with androgen deprivation or castration therapies (surgical or medical) based on the findings reported about 80-years ago. However, this androgen removal approach is not curative for prostate cancers, and the diseases often relapse and progress to the CRPC stage. Since most of these CRPCs are still AR addictive, current clinical therapies mainly focus on blocking androgen to bind with the AR LBD (AR antagonists) or reducing androgen production (CYP17a1 inhibitors) in non-testis tissues, including prostate cancer tissues. However, treatment resistance eventually develops in part due to AR gene mutation and mRNA splicing events (e.g., AR-V7) in virtually all CRPC patients. Furthermore, after long-term treatment with AR antagonists, up to 20% of CRPC patients will develop an even more aggressive subtype, neuroendocrinal prostate cancer (NEPC). Therefore, the androgen removal and blockage approach are non-curative and leads to a more aggressive disease.

To overcome this obstacle of treatment resistance, research has shifted from androgens to the AR protein in the last 20 years (**Figure 1**). The initial approach was the antisense oligonucleotides (ASO) targeting the AR mRNA to reduce AR protein production in prostate cancer cells. Due to the inhibitory nature of the ASO approach on protein production, tumor growth was only suppressed but not eradicated in xenograft models. In contrast, our group used the siRNA approach that efficiently eliminated the AR protein from prostate cancer cells. Nanoparticle-loaded AR siRNA resulted in xenograft tumor regression and eradication owing to robust cell death after AR protein removal in prostate cancer cells. Unfortunately, this AR siRNA project was stalled due to a failure in the patent application.

Targeting AR protein stability has emerged in recent years as the hotspot in developing new therapeutics for advanced prostate cancers, and several small molecules were reported to reduce AR protein stability. The curcumin analog ASC-J9, Ailanthone, HG122, and CUDC-101 induced AR protein degradation in prostate cancer cells. However, the AR or prostate cancer tissue specificity is not established with these small molecules. The PROTAC technique for AR-specific degradation showed a promising result. The AR PROTAC ARV-110 is tested as a combinational treatment with Abiraterone in a clinical trial. However, these AR CTD-targeting PROTACs utilized AR LBD ligands, and therefore, they are inactive on AR CTD splicing variants, a critical mechanism for treatment resistance in CRPC patients. Interestingly, some other agents specifically targeted the AR-V7 variant for degradation, including Niclosamide, CUDC-101, Thailanstatins, Rutaecarpine, Indisulam, and Nobiletin. Combining AR antagonists, PROTAC molecules, and AR-V7 inhibitors might provide synergistic effects in the clinic.

Targeting AR NTD is another approach to bypass AR CTD splicing defects. The first generation of AR NTD inhibitor EPI compounds was failed in clinical trials due to excessive bill burden. The second generation of EPI compound with 20-fold higher potency is being tested as a combinational treatment with Enzalutamide in a clinical trial. UT-34 targets the AR NTD and is also waiting for a clinical test.

AR nuclear translocation is an important event for its activity as a transcription factor. Two novel compounds, IMPPE and JJ-450, were recently developed to block AR nuclear translocation. These two compounds showed a very permissive result in animal models. In addition, an AR DBD blocking agent VPC-14449 was reported to suppress AR interaction with its target gene promoter in the nuclear compartment and was found to suppress tumor growth in mice. These compounds are all needed for clinical testing.

AR activity is only temporally suppressed during prostate cancer treatment by androgen deprivation and AR antagonists. Due to these treatment stresses, prostate cancer cells used other cellular signal pathways and/or splicing variants for AR reactivation, resulting in treatment resistance. Therefore, complete removal of the AR protein from prostate cancer cells will eliminate all events of AR reactivation after ADT and anti-AR therapy. Especially in the early phase of treatment, most prostate cancer cells are still AR-dependent. Simultaneously removal of the AR protein and androgens will result in robust cell death, leading to a possible curative result or long-term disease-free survival. In addition, early reduction of the AR protein in the androgen-responsive phase of prostate cancer will reduce the likelihood of transcriptional reprogramming (88, 93, 147). Also, tissue-specific delivery of the AR protein degradation agents will restrict potential side effects.

## Author Contributions

All authors participated in drafting the manuscript. All authors contributed to the article and approved the submitted version.

## Funding

This work was partially supported by a grant from KUMC Lied pilot program and DoD PCRP PC190026 to Benyi Li, MD/PhD.

## Conflict of Interest

The authors declare that the research was conducted in the absence of any commercial or financial relationships that could be construed as a potential conflict of interest.

## Publisher’s Note

All claims expressed in this article are solely those of the authors and do not necessarily represent those of their affiliated organizations, or those of the publisher, the editors and the reviewers. Any product that may be evaluated in this article, or claim that may be made by its manufacturer, is not guaranteed or endorsed by the publisher.
